# Molecular diagnosis of patients with syndromic short stature identified by trio whole-exome sequencing

**DOI:** 10.3389/fgene.2024.1399186

**Published:** 2024-10-02

**Authors:** Huihui Sun, Geng Zhang, Na Li, Xiangfang Bu

**Affiliations:** ^1^ Department of Paediatrics, Beijing Jishuitan Hospital, Capital Medical University, Beijing, China; ^2^ Beijing Chigene Translational Medical Research Center Company, Beijing, China; ^3^ Department of Radiology, Beijing Jishuitan Hospital, Capital Medical University, Beijing, China

**Keywords:** short stature, molecular diagnosis, trio whole-exome sequencing, variant, uniparental disomy

## Abstract

**Background:**

Short stature is a complex disorder with phenotypic and genetic heterogeneity. This study aimed to investigate clinical phenotypes and molecular basis of a cohort of patients with short stature.

**Methods:**

Trio whole-exome sequencing (Trio-WES) was performed to explore the genetic aetiology and obtain a molecular diagnosis in twenty Chinese probands with syndromic and isolated short stature.

**Results:**

Of the twenty probands, six (6/20, 30%) patients with syndromic short stature obtained a molecular diagnosis. One novel *COMP* pathogenic variant c.1359delC, p.N453fs*62 and one *LZTR1* likely pathogenic variant c.509G>A, p.R170Q were identified in a patient with short stature and skeletal dysplasia. One novel *de novo NAA15* pathogenic variant c.63T>G, p.Y21X and one novel *de novo KMT2A* pathogenic variant c.3516T>A, p.N1172K was identified in two probands with short stature, intellectual disability and abnormal behaviours, respectively. One patient with short stature, cataract, and muscle weakness had a *de novo POLG* pathogenic variant c.2863 T>C, p.Y955H. One *PHEX* pathogenic variant c.1104G>A, p.W368X was identified in a patient with short stature and rickets. Maternal uniparental disomy 7 (mUPD7) was pathogenic in a patient with pre and postnatal growth retardation, wide forehead, triangular face, micrognathia and clinodactyly. Thirteen patients with isolated short stature had negative results.

**Conclusion:**

Trio-WES is an important strategy for identifying genetic variants and UPD in patients with syndromic short stature, in which dual genetic variants are existent in some individuals. It is important to differentiate between syndromic and isolated short stature. Genetic testing has a high yield for syndromic patients but low for isolated patients.

## Introduction

Short stature is a common disorder for referral to paediatric endocrinologists (Collett-Solberg et al., 2019a). The clinical diagnosis of short stature is challenging based mainly on birth weight, growth pattern, clinical manifestations, family history, and laboratory tests. It is important to differentiate between isolated short stature and syndromic short stature, which is concurrent with facial dysmorphism, skeletal dysplasia and other malformations (Jee et al., 2018). Nevertheless, some clinical manifestations are unspecific and mild, especially in young children. With the rapid development of molecular technology, recent studies have demonstrated that the disorder is mainly caused by various genetic aberrations, including polygenic inheritance, chromosomal abnormalities, copy number variants (CNVs), uniparental disomy of chromosome (UPD), nuclear genetic variants, mitochondrial genetic variants, and methylation defects (Wit et al., 2016; Grunauer and Jorge, 2018; Homma et al., 2018). Treatment with recombinant human growth hormone (rhGH) is an effective approach for improving heights of children with short stature; however, not all of these patients can be treated with rhGH, and neither the patient’s response to rhGH nor the adverse effects in the context of some genetic disorders are well understood (Murray et al., 2018; Collett-Solberg et al., 2019a).

A definitive molecular diagnosis provides important information about specific associated features, prognostic predictions, individualized therapeutic options and genetic counselling (Jee et al., 2018). Recent studies have demonstrated that isolated and syndromic short stature could be caused by monogenic defects in the growth hormone insulin-like growth factor 1 (GH-IGF-1) axis, cartilage growth plate, fundamental cellular processes and signalling pathway (Hu et al., 2016; Andrade et al., 2017; Homma et al., 2019; Stavber et al., 2022). Trio whole-exome sequencing (Trio-WES) is a diagnostic approach for identifying genetic variations in patients with syndromic short stature with unknown aetiology (Sun et al., 2021). Despite tremendous advances in the molecular genetics of growth disorders, the underlying molecular mechanisms remain unknown in a significant proportion of individuals with short stature (Zahnleiter et al., 2013). In this study, Trio-WES was performed to explore genetic causes in patients with isolated short stature and syndromic short stature (Miller et al., 2010; Wang et al., 2020). We describe detailed clinical manifestations and molecular analysis in these patients to guide individualized therapy.

## Methods

### Patients

This study was approved by Ethics Committee of Beijing Jishuitan Hospital, Capital Medical University (202104-07 and 202201-20-01). Patients were referred to the paediatric department of Beijing Jishuitan Hospital, Capital Medical University from January 2021 to December 2022. The diagnostic criteria of short stature were as follows (Siklar and Berberoglu, 2014; Grunauer and Jorge, 2018; Collett-Solberg et al., 2019b): height or predicted adult height (PAH) measuring 1.88 standard deviations (SD) below the mean for individuals of the same age and sex among healthy Chinese children aged 1–15 years. The exclusion criteria were chronic systematic diseases and comorbidities that could affect growth, such as malnutrition, psychological disturbances, hypothyroidism, and known syndromes. Patients were classified into two groups: isolated short stature and syndromic short stature, which was concurrent with multiple malformations including facial dysmorphism, intellectual disability, skeletal dysplasia and other abnormalities (Collett-Solberg et al., 2019b). Twenty Chinese trios of probands with short stature were recruited in the study. Written informed consent was obtained from their legal guardians and parents.

### Trio-WES

Genomic DNA was extracted from peripheral blood lymphocytes of the probands and their available family members using the Blood Genome Column Medium Extraction Kit (Kangweishiji, China). Trio-WES was performed in the entire cohort by Beijing Chigene Translational Medical Research Center. Exome capture was carried out using xGen Exome Research Panel v2.0 (IDT, Iowa, United States), which consists of 429,826 individually synthesized and quality-controlled probes, targeting a 39 Mb protein-coding region (19,396 genes) of the human genome and covering 51 Mb of end to-end tiled probe space. High-throughput sequencing (100×) was performed by MGI DNBSEQ-T7 sequencing instruments (PE150) and the average coverage of target sequence was above 99%.The online system independently developed by Chigene (www.chigene.org) was used to annotate database-based minor allele frequencies (MAFs) and American college of medical genetics (ACMG) practice guideline-based pathogenicity of genetic variants. Bioinformatics software tools, including VarCards (http://varcards.biols.ac.cn/) and InterVar (https://wintervar.wglab.org/), were both applied for clinical interpretation of each genetic variant according to the ACMG and the Association for Molecular Pathology (ACMG/AMP) 2015 guidelines (Richards et al., 2015). Genotype-phenotype correlations were analysed by crossreferencing with Online Mendelian Inheritance in Man (OMIM, https://www.omim.org/), ClinVar databases and the relevant references. The identified genetic variants were classified as “benign”, “likely benign”, “variants of uncertain significance (VUS)”, “likely pathogenic” or “pathogenic”. Sanger sequencing was further used to confirm the candidate variants.

## Results

Clinical features and Trio-WES results of twenty probands are summarized in Table 1. In this cohort, thirteen patients (13/20, 65%) were diagnosed as isolated short stature including twelve patients with idiopathic short stature (ISS) and one patient born small for gestational age (SGA). Seven probands (7/20, 35%) including one with SGA were diagnosed with syndromic short stature. These abnormal features included skeletal dysplasia (n = 6, P4, P6, P8, P9, P14, and P19), facial dysmorphism (n = 4, P4, P8, P9, and P19), intellectual disability (n = 2, P8, and P9), and visual impairment (n = 2, P9, and P13).

**TABLE 1:** Clinical phenotypes and genetic analysis in patients with short stature.

Patient	Year	Sex	Height (cm,SD)	IGF-1 (ng/mL)	BA (years)	Intellectual disability	Facial dysmorphism	Other clinical features	Sequencing results	Diagnosis
1	1	Male	68.4, −3.02	26.4 (16–134)	—	—	—	—	*SMAD2*, NM_005901, c.683A>C, p.E228A (VUS)	ISS
2	5.25	Male	104.7, −1.96	79 (50–286)	4	—	—	—	—	ISS
3	4.58	Female	99.3, −2.00	114.33 (49–283)	3	—	—	—	—	ISS
4	7.42	Male	116.5, −2.01	120.35 (57–316)	5.5	—	Macrocephaly, wide forehead, epicanthal folds	Pectus deformity, flat foot	*COMP*, NM_000095.3, c.1359delC, p.N453fs*62; *LZTR1*, NM_006767.4, c.509G>A, p.R170Q	syndromic short stature
5	8.75	Female	131.0, −0.32 (PAH: 150.35, −1.90)	234 (64–345)	10.5	—	—	—	*FGFR2*, NM_022970.3, c.1378A>G, p.M460V (VUS)	ISS
6	11.25	Female	135.4, −1.95	257.55 (111–551)	10	—	—	Horseshoe kidney, perineal fistula, strabismus, bilateral thoracic asymmetry, subclinical hypothyroidsm	*DNMT3A*, NM_175629, c. 28G>A, p.G10R (VUS)	syndromic short stature
7	7.92	Female	116.8, −2.20	146.0 (57–277)	8	—	—	—	—	ISS
8	9.92	Male	118.4, −3.59	74.31 (40–255)	7	+	+	Syndactyly, ASD	*NAA15*, NM_057175.5, c.63T>G p.Y21X	syndromic short stature
9	3.58	Male	93.2, −2.18	60.31 (15–129)	3.5	+	+	Hypertrichosis, pectus deformity, abnormal behavior	*KMT2A*, NM_001197104.2, c.3516T>A, p.N1172K	syndromic short stature
10	9.67	Male	134.8, −0.68 (PAH: 158.5, −2.33)	122.97 (40–225)	12	—	—	Obesity, advanced bone age	—	ISS
11	5.33	Female	102.6, −2.22	108.68 (35-232)	5	—	—	Birth Weight 2250 g at 39 weeks	*NPR2*, NM_003995, c.1815 + 12 (IVS11) G>A (VUS)	ISS, SGA
12	3.25	Male	90.7, −2.26	41.62 (15–129)	2	—	—	—	*TONSL*, NM_013432, c.10G>C, p.E4Q (VUS)	ISS
13	3	Male	87.4, −2.67	48.83 (15–129)	3	—	—	Sparse hair, cataracts, muscle weakness	*POLG*, NM_002693.3, c.2863T>C, p.Y955H	syndromic short stature
14	10.42	Male	116.3, −4.17	162.05 (69–316)	9	—	—	Rickets	*PHEX*, NM_000444.6, c.1104G>A, p.W368X	syndromic short stature
15	3.92	Female	90.9, −3.16	108.78 (18–172)	4	—	—	—	*ARX*, loss1(EXON4-5), chrX:25022786-25025556*1	ISS
16	3.17	Male	88.2, −2.76	68.60 (15–129)	3	—	—	—	*AFF4*, NM_014423, c.2053G>A, p.D685N (VUS)	ISS
17	9.92	Male	126.4, −2.21	113.14 (40–225)	7	—	—	—	*GPR161*, NM_001267609, c.748G>A, p.V250M (VUS)	ISS
18	7.83	Male	116.1, −2.37	73.41 (40–225)	5	—	—	—	*DAX1*, NM000475.5, c.956A>T, K319M (VUS); *IGF1R*, NM_000875.5, c.3769C>T, p.P1257S (VUS)	ISS
19	3.75	Male	82.8, −5.24	32.7 (30–155)	2.5	—	Triangular face, prominent forehead	Clinodactyly V	mUPD7	syndromic short stature, SGA
20	14.83	Male	151.1, −2.78	459 (177–507)	14	—	—	—	—	ISS

Of the twenty probands, six (6/20, 30%) patients with syndromic short stature obtained molecular diagnosis. Thirteen patients with isolated short stature had negative results. One novel *COMP* pathogenic variant c.1359delC, p.N453fs*62 and one *LZTR1* likely pathogenic variant c.509G>A, p.R170Q were identified in a patient with short stature and skeletal dysplasia. One novel *de novo NAA15* pathogenic variant c.63T>G, p.Y21X and one novel *de novo KMT2A* pathogenic variant c.3516T>A, p.N1172K was identified in two probands with short stature, intellectual disability and abnormal behaviours, respectively. One patient with short stature, cataract, and muscle weakness had a *de novo POLG* pathogenic variant c.2863 T>C, p.Y955H. One *PHEX* pathogenic variant c.1104G>A, p.W368X was identified in a patient with short stature and rickets. The primers used by Sanger sequencing to confirm these variants are shown in the Supplementary Table. Maternal uniparental disomy 7 (mUPD7) was pathogenic in a patient with pre and postnatal growth retardation, wide forehead, triangular face, micrognathia and clinodactyly. Clinical manifestations of these patients are described in detail sequentially below.

### *COMP* variant and *LZTR1* variant

**FIGURE 1:**
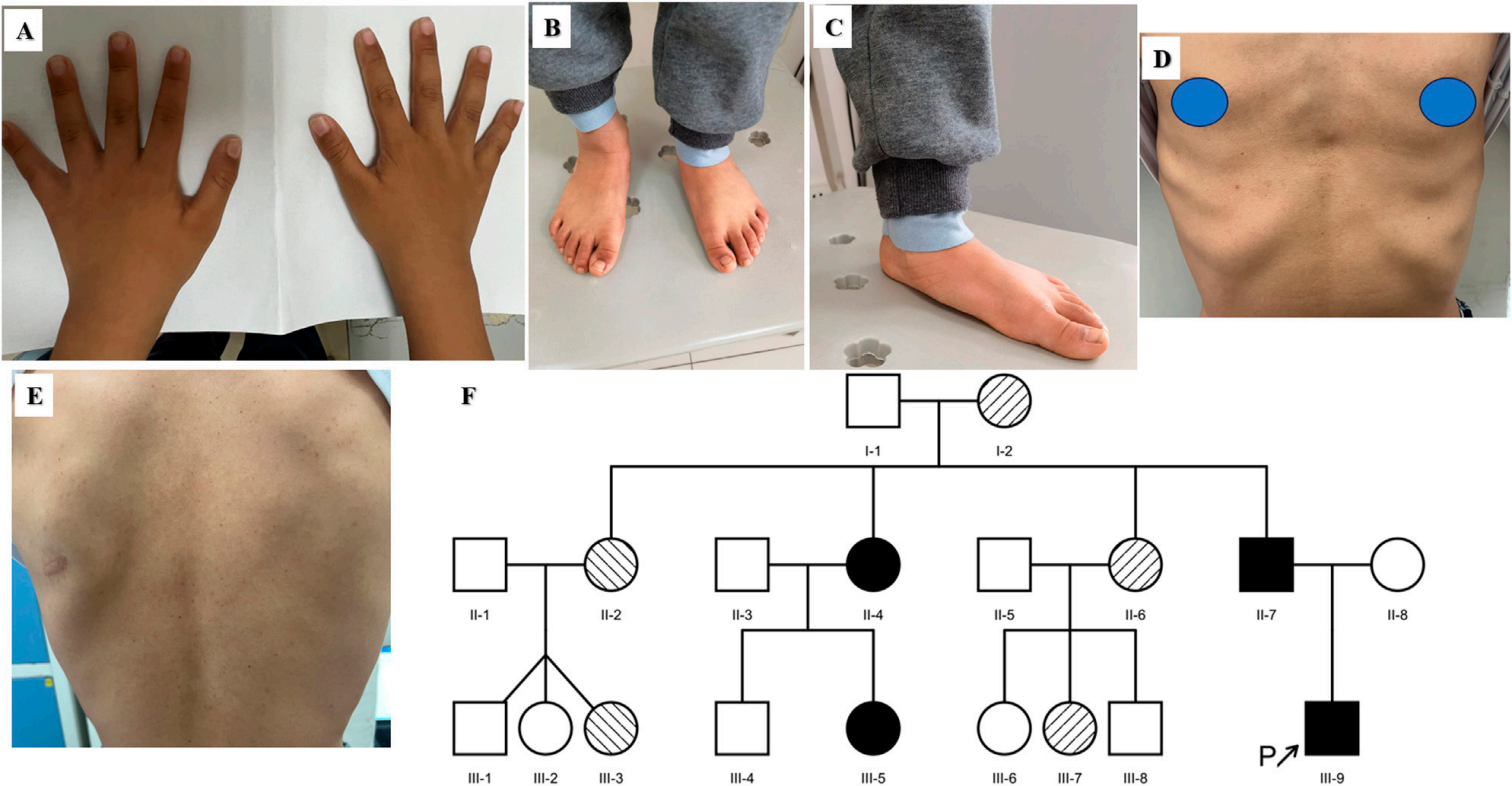
Clinical features of the fourth proband **(A–C)** and his father **(D, E)**. **(A)** and **(B)** Wrinkled skin of the hands and feet. **(C)** Flatfoot. **(D)** Pectus deformity. **(E)** Multiple lentigines of the trunk. **(F)** Pedigree tree of the fourth proband. The black arrow represents the proband. The shaded symbols represent the affected individuals. The members I-2, II-6 and III-7 had the *COMP* variant (Right diagonal). The members II-2 and III-3 had the *LZTR1* variant (Left diagonal). The members II-4, II-7, III-5 and III-9 had both the *COMP* variant and *LZTR1* variant (Solid). The members II-3, II-8 and III-4 were normal. Blood sample of other members were unavailable.

The fourth proband was a boy aged 7 years and 5 months. He showed signs of short stature after 2 years of age. The patient was born at full term by caesarean section. His birth weight was 3,600 g, and his birth length was 50 cm. He had no history of perinatal asphyxia. His height was 116.5 cm (−2.01 SD), weight 21.2 kg (−1.24 SD), and BMI 15.62 kg/m^2^ (−0.08 SD) at visit. He had normal intelligence. His head circumference was 54 cm. The upper segment was 62.5 cm, and lower segment was 54.0 cm. The upper/lower segment ratio was 1.16 (−0.82 SD). His arm span was 114 cm. The proband had macrocephaly, wide forehead, epicanthal folds, pectus deformity, wrinkled skin on the hands and feet, flat foot and café-au-lait skin pigmentation (Figures 1A–C). He had no short neck or webbed neck. Growth hormone stimulation tests showed peak concentrations of 8.14 ng/mL after combined treatment with levodopa and arginine. The proband had normal liver and kidney function, myocardial enzyme levels and thyroid function. The echocardiography and urological ultrasound findings were all normal. His bone age (BA) was 5.5 years, 2 years below his chronological age (CA). His father’s height, weight and BMI were 164.4 cm (−1.36 SD), 52.4 kg, and 19.39 kg/m^2^, respectively. He had facial dysmorphism, including a triangular face and prominent nasolabial folds. He had pectus deformity (Figure 1D), movement limitation of the lumber spine, and flatfoot. He had multiple lentigines on the face and trunk (Figure 1E), and wrinkled skin on the hands and feet. He had complained of ankle pain after long-distance walking beginning at the age of 30 years. The height, weight and BMI of the proband’s mother were 154.7 cm, 58.8 kg, and 24.57 kg/m^2^, respectively. She had no complaints. A *COMP* pathogenic variant c.1359delC p.N453fs*62 in exon 13 was identified in the proband and his father. There were five other members segregating with this variant including I-2 (153 cm, −1.41 SD), II-4 (154 cm, −1.22 SD), II-6 (155 cm, −1.04 SD), III-5 (123 cm, 0.04 SD), and III-7 (120 cm, −1.27 SD) confirmed by sanger sequencing. The pedigree tree is shown in Figure 1F. All adult members carrying the *COMP* variation had short stature or low–normal height and joint pain, including in the knees, wrist, ankle, and lumber vertebra. There was no complaint of joint pain among the young affected members (III-5, III-7 and III-9). A *LZTR1* heterozygous variant c.509G>A p.R170Q (MAF 0.000014/2, GnomAD) in exon five was identified in the proband and his father. There were four other affected members with this variant, including II-2 (152 cm, −1.59 SD), III-3 (152 cm, −0.63 SD), II-4 (154 cm, −1.22 SD), and III-5 (123 cm, 0.04 SD). *In silico* analysis predicted that it was a likely pathogenic variant (InterVar, PM1, PM2, PP3 and PP5) according to clinical interpretation of genetic variants by ACMG/AMP 2015 guideline. Blood sample of the members I-1, II-1, II-5, III-1, III-2, III-6, and III-8 were unavailable, preventing molecular testing.

### NAA15

**FIGURE 2:**
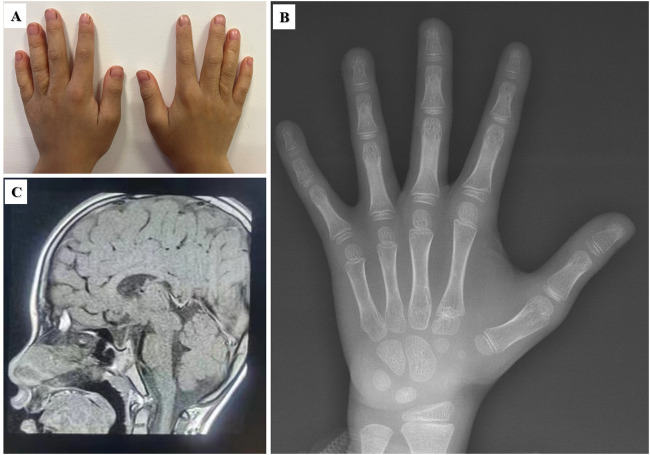
Clinical features of the eighth proband with the *NAA15* variant. **(A)** Surgical scar of partial syndactyly in both hands. **(B)** Bone age radiograph. CA: 9/11/12 years; BA: 7 years **(C)** Partial empty sella of the pituitary gland.

The eighth proband was a boy aged 9 years and 11 months. He showed signs of short stature. The patient was born at full term by vaginal delivery. His birth weight was 2,690 g, and his birth length was 49 cm. He had no history of perinatal asphyxia. His height, weight and BMI were 118.4 cm (−3.59 SD), 18.55 kg (−3.35 SD), and 13.23 kg/m^2^ (−2.14 SD), respectively. He had intellectual disability and language delay. He was diagnosed with autism spectrum disorder (ASD) at 5 years old. The proband had prominent eyebrows, epicanthus inversus, anteverted nares, multiple dental caries and low-set and posteriorly rotated ears. He had partial syndactyly between the third and fourth fingers in both hands since birth and had completed surgical management (Figure 2A). His growth hormone stimulation tests showed peak concentrations of 14.20 ng/mL after combination treatment with levodopa and arginine. His BA was 7 years, which was 3 years below CA (Figure 2B). His pituitary MRI showed a partial empty sella (Figure 2C). One novel *de novo NAA15* variant c.63T>G, p.Y21X was identified in the proband.

### KMT2A

**FIGURE 3:**
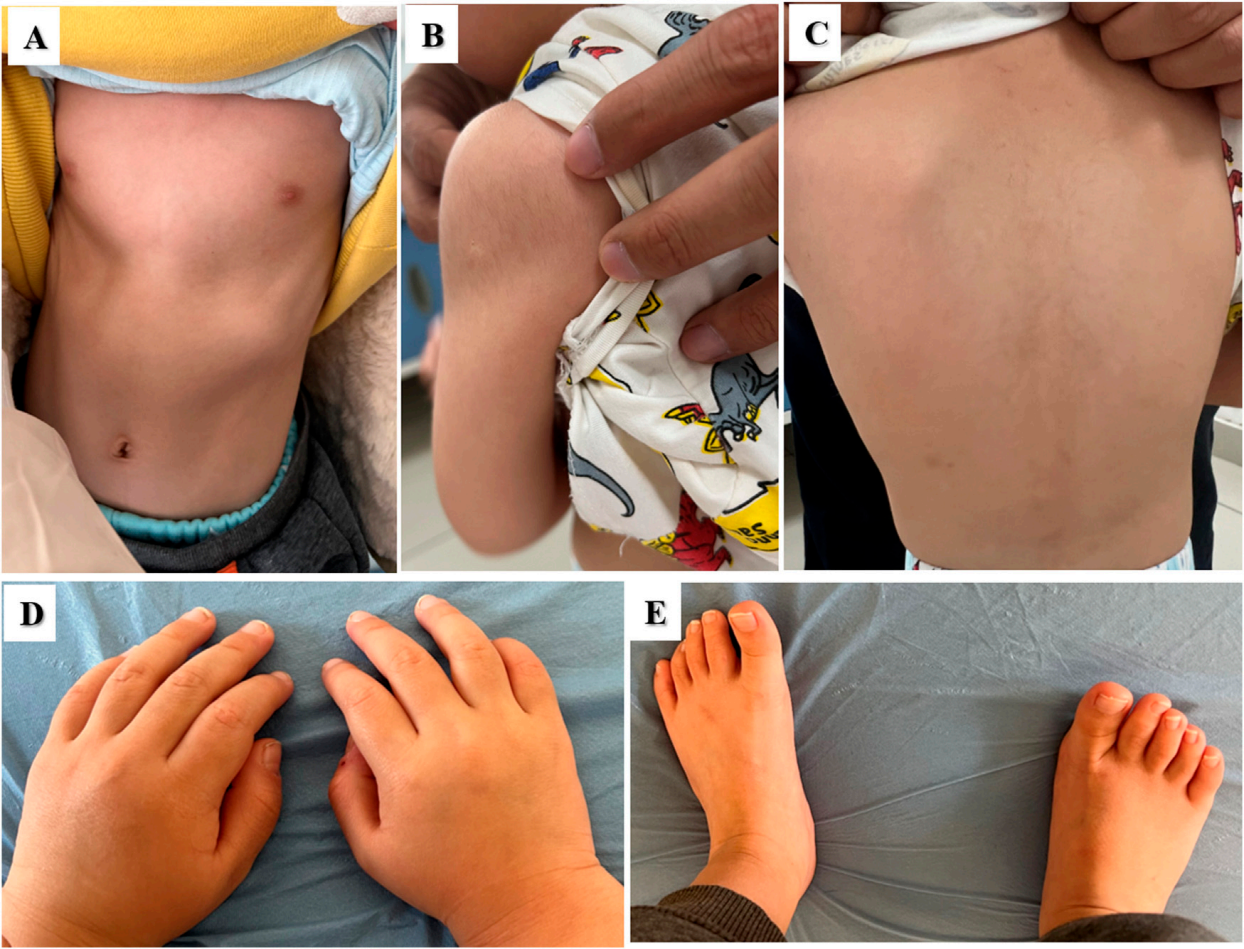
Clinical features of the nineth proband with *KMT2A* variant. **(A)** Pectus deformity. **(B)** and **(C)** Hypertrichosis on the upper arms and back. **(D, E)** Puffy hands and feet.

The ninth proband was a boy aged 3 years and 7 months. He showed signs of short stature. The patient was born at full term by vaginal delivery. His birth weight was 3,850 g, and his birth length was 47 cm. He had no history of perinatal asphyxia. His height, weight and BMI were 93.2 cm (−2.18 SD), 12.1 kg (−2.51 SD), and 13.93 kg/m^2^ (−1.41 SD), respectively. His head circumference was 47 cm (−2.40 SD). He had intellectual disability, language delay, and abnormal hyperactivity behaviours. The proband had an abnormal facial appearance: flat face, thick eyebrows, epicanthus, ptosis, long eyelashes, downslanting and vertically narrow palpebral fissures, strabismus, wide nasal bridge, broad nasal tip and thin upper vermillion border. He had slim build, muscular hypotonia, pectus deformity, hypertrichosis on the upper arms and back, puffy hands and feet (Figure 3). He had chronic constipation and poor sleep since birth. His echocardiography and urological ultrasound were normal. His BA was 3.5 years. His father’s height was 176 cm, and his mother’s height was 160 cm, and their phenotypes were normal. Trio-WES identified a *KMT2A de novo* variant c.3516T>A, p.N1172K in the proband.

### POLG

The thirteenth proband was a male patient aged 3 years. The child was born by breech delivery at term. His birth weight was 2,950 g. After birth, he had hypoxia asphyxia. At visit, his height, weight and BMI were 87.4 cm (−2.67 SD), 10.7 kg (−3.01 SD), and BMI 14.01 kg/m^2^ (−1.54SD), respectively. The proband had normal thyroid function, liver and kidney function, myocardial enzymes and blood electrolytes. His BA was equivalent to 3 years. Then he was diagnosed as isolated short stature. At the follow-up of 4 years and 1 month, he was diagnosed with bilateral cataracts in ophthalmic department. Then his height, weight and BMI were 91.9 cm (−3.29 SD), 11.95 kg (−3.14 SD), and BMI 14.15 kg/m^2^ (−1.06SD), respectively. His upper segment was 48.9 cm and his lower segment was 43.0 cm. The upper/lower segment ratio was 1.13 (−3.61 SD). His head circumference was 50 cm (−0.27 SD). Muscle weakness and exercise intolerance were apparent. His BA was about 4 years. He had elevated liver and muscle enzymes including AST 50.8 (14–44) U/L, GGT 19.4 (5–19) U/L, CG 4.48 (0–2.70) mg/L, ADA 26.3 (0–25) U/L, LDH 331 (110–295) U/L, and HBDH 283 (80–220) U/L. He had high TBA 13.48 (0–10) μmol/L, high TG 3.86 (0.4–1.7) mmol/L and low HDL 0.9 (1–1.55) mmol/L levels. A *de novo POLG* pathogenic variant c.2863T>C, p.Y955H was identified in the proband.

### PHEX

**FIGURE 4:**
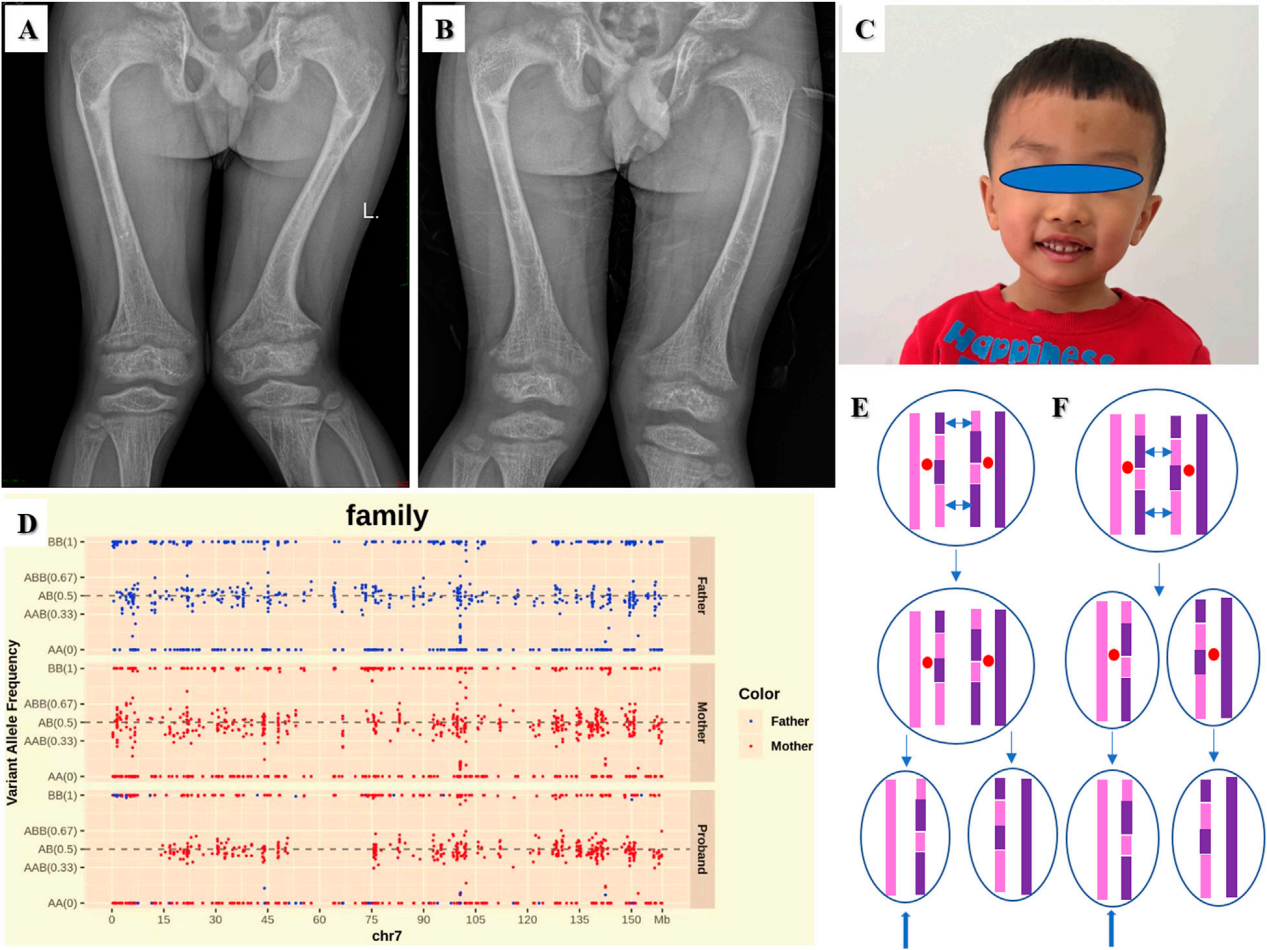
Radiographic features of the fourteenth proband **(A)** and his younger brother **(B)**: frayed and cupped metaphyses, pseudofractures, and osteoporosis in the femurs. **(C)** The facial feature of the nineteenth proband. **(D)** SNPs analysis based on Trio-WES revealed a mixture of maternal isodisomy and heterodisomy of chromosome 7. The possible mechanism of the mixture pattern of uniparental disomy (UPD) is based on recombination of maternal homologs and maternal nondisjunction at Meiosis I **(E)** or Meiosis II **(F)**. The double-headed arrows show crossing over of genetic material between homologous chromosomes during meiosis. The blue arrows show a mixture of maternal isodisomy and heterodisomy of chromosome 7.

The fourteenth proband was a male patient aged 10 years and 10 months. He showed signs of rickets. He manifested with severe lower extremity bowing, impaired mobility, and genu valgus. His height, weight and BMI were 116.3 cm (−4.39 SD), 24.3 kg (−2.23 SD), and 17.97 kg/m^2^ (0.20 SD), respectively. His upper segment was 67.0 cm (−2.70 SD), and his lower segment was 49.3 cm. The upper/lower segment ratio was 1.36 (4.04 SD). His pubertal stage was Tanner II. The child was born by caesarean section at 34 weeks’ gestational age. His birth weight was 2,800 g. The proband had normal liver and kidney function, myocardial enzymes and blood electrolytes. The findings for the indicators of bone metabolism were as follows: Ca 2.26 (2.25–2.67) mmol/L, P 0.67 (1.45–2.10) mmol/L, and ALP 673 (50–400) IU/L. His intact parathyroid hormone (iPTH) level was 63.9 (15–65) pg/mL. He had a low level of 25-(OH)-VD_3_ 17.77 (30–100) ng/mL. His BA was equivalent to 9 years. He had normal thyroid function. His radiographic features were widened, frayed, and cupped metaphyses, pseudofractures, and osteoporosis in the femurs (Figure 4A). The younger brother of the proband was 7 years and 3 months old. His manifestations and radiographic features (Figure 4B) were similar to the proband. His height, weight and BMI were 105.1 cm (−4.16 SD), 18.9 kg (−1.95 SD), and 17.11 kg/m^2^ (0.77 SD), respectively. His upper segment was 63.5 cm (−1.95 SD), and his lower segment was 41.6 cm. The upper/lower segment ratio was 1.53 (5.48 SD). Bilateral cryptorchidism was present at birth. The findings for the indicators of bone metabolism were as follows: Ca 2.21 (2.25–2.67) mmol/L, P 0.58 (1.45–2.10) mmol/L, and ALP 870 (50–400) IU/L. His iPTH was 93.9 (15–65) pg/mL. He had a low level of 25-(OH)-VD_3_ 18.16 (30–100) ng/mL. His BA was equivalent to 6 years. His father had a normal phenotype with a height of 170 cm. His mother had short stature and rickets. She had received surgical management. Her height was 141.4 cm (−3.55 SD). A *PHEX* pathogenic variant c.1104G>A, p.W368X in exon 10 was identified in the proband, his younger brother, and their mother.

### UPD 7

The nineteenth proband was a boy aged 3 years and 9 months. The patient was born at full term by caesarean section. His birth weight was 2,100 g, and his birth length was 46 cm. He was diagnosed as SGA. He had feeding difficulties after birth. At visit his height was 82.8 cm (−5.24 SD), weight 8.8 kg (−5.24 SD), and BMI 12.84 kg/m^2^ (−2.62 SD). His head circumference was 46.2 cm (−2.90 SD). His upper segment was 44.8 cm, and his lower segment was 38.0 cm. The upper/lower segment ratio was 1.18 (−3.11SD). His arm span was 83.0 cm. The proband had slim build, wide forehead, triangular face, micrognathia and fifth-finger clinodactyly (Figure 4C). Growth hormone stimulation tests by levodopa showed peak concentrations of 13.03 ng/mL. The proband had normal liver and kidney function, myocardial enzyme levels and thyroid function. The echocardiography and urological ultrasound findings were normal. His BA was 2.5 years. His father’s and mother’s height was175 cm and 153 cm, respectively. Single nucleotide polymorphisms (SNPs) analysis based on Trio-WES revealed mUPD 7, maternal isodisomy at chr7:1-12727826 and chr7:63505837-75513216 and maternal heterodisomy at chr7:12727826-63505837 and chr7: 75513216-159138663 as shown in Figure 4D. The possible mechanism is based on recombination of maternal homologs and maternal nondisjunction at Meiosis I (Figure 4E) or Meiosis II (Figure 4F).

## Discussion

We performed Trio-WES in a cohort of Chinese children with syndromic and isolated short stature. The diagnostic yield in the entire cohort was 30% (6/20), which was consistent with previous studies (Huang et al., 2018; Murray et al., 2018). Six pathogenic/likely pathogenic genetic variants were identified in five patients with syndromic short stature, including four monogenic pathogenic variants and one patient with dual pathogenic and likely pathogenic variants. Importantly, mUPD7 was identified in a patient with SGA and dysmorphic features.

Cartilage oligomeric matrix protein encoded by *COMP* (MIM *600310) is an extracellular matrix (ECM) glycoprotein and plays an important role in the structural integrity of cartilage and chondrocyte growth plate development (Posey et al., 2018; Caron et al., 2020). It has been demonstrated that *COMP* variants are associated with multiple epiphyseal dysplasia (MED, MIM#132400) and pseudoachondroplasia (PSACH, MIM #177170) with autosomal dominant inheritance (El-Lababidi et al., 2020). *COMP* variants could result in endoplasmic reticulum retention of COMP in chondrocytes and negative regulation of cartilage growth (Posey et al., 2018). MED is a genetic skeletal dysplasia characterized by delayed and irregular ossification of the epiphyses and early-onset osteoarthritis (Shao et al., 2020). The *COMP* pathogenic variant p.N453fs*62 segregated with short stature or low-normal height and early-onset osteoarthritis in the three-generation pedigree. The proband had short stature and delayed BA maturation. His father had low–normal height, movement limitation of the lumbar spine, and joint pain. All adult members carrying the *COMP* variation had a short height and various degrees of joint pain. This suggests that patients with short stature, delayed BA and a family history of early arthritis may have a *COMP* variant. Additionally, a *LZTR1* likely pathogenic variant p.R170Q was identified in the proband. *LZTR1* encoding leucine-zipper-like transcription regulator 1, which belongs to the BTB-POZ protein superfamily, is a tumour suppressor gene (Jacquinet et al., 2020). The variant p.R170Q localized in the KELCH 2 domain has been predicted to enhance RAS and MAPK signalling and identified in patients with Noonan syndrome and schwannomatosis (Piotrowski et al., 2014; Yamamoto et al., 2015; Johnston et al., 2018; Motta et al., 2019). Noonan syndrome (MIM #616564) called RASopathy syndrome is characterized by craniofacial malformations, cardiac abnormalities, short stature, cryptorchidism, and cognitive impairment (Roberts, 1993; Roberts et al., 2013; Noonan and Kappelgaard, 2015). The proband harbouring *LZRT1* variant was characterized by short stature, facial abnormalities, and pectus deformity. His father had typical facial features, pectus deformity, and multiple lentigines. In a patient with Noonan syndrome carrying the *LZTR1* variant c.850C>T, p.Arg284Cys, there were possible associations between rhGH treatment and late-onset oligoastrocytoma and ganglioblastoma (Jacquinet et al., 2020). We used rhGH in the proband with caution due to risks of nervous system disorders associated with *LZTR1* variants (Piotrowski et al., 2014; Jacquinet et al., 2020). Genotype-phenotype analysis suggested that clinical manifestations of the proband and his father had features of dual disorders of MED and Noonan syndrome. The findings allowed us to identify a novel *COMP* pathogenic variant and a *LZTR1* likely pathogenic variant segregating in the pedigree.

*NAA15*, located at 4q31.1, encodes for N-alpha-acetyltransferase 15, which is the auxiliary subunit of the N-terminal acetyltransferase (NatA) complex that is crucial for posttranslational modification of proteins (Stessman et al., 2017). NAA15 plays an important role in the generation and differentiation of neurons in development of the nervous system (Cheng et al., 2018; Zhao et al., 2018). This deleterious variant p.Y21X identified in our study is predicted to induce nonsense-mediated decay (NMD) and haploinsufficiency, affecting either NatA co-translational activity or complex stability (Cheng et al., 2018; Cheng et al., 2019; Lyon et al., 2023). *NAA15* (OMIM *608000) variants have been identified in patients with intellectual disability, speech delay, ASD, facial dysmorphology and cardiac anomalies (MIM # 617787) (Stessman et al., 2017; Cheng et al., 2018). Clinical manifestations of the proband included short stature, intellectual disability, speech delay, ASD, facial abnormalities, and syndactyly. He had a partial empty sella but no growth hormone deficiency. In contrast to previous studies, we highlight the vital role of *NAA15* in growth development based on short stature in the proband and the mechanism requires further functional study (Cheng et al., 2018; Zhao et al., 2018). The proband had a good response to rhGH during the follow-up period. Other pituitary hormones should be monitored (Ucciferro and Anastasopoulou, 2022).

*KMT2A* (OMIM *159555) encodes a DNA-binding histone methyltransferase that regulates chromatin-mediated transcription through histone H3K4 methylation and positively regulates expression of target genes (Milne et al., 2002). It has been identified as the pathogenic gene of Weidemann-Steiner syndrome (WSS, MIM #605130) (Jones et al., 2012). We identified a novel *de novo KMT2A* likely pathogenic variant p. N1172K in the proband with short stature, intellectual disability, facial malformations, ocular abnormalities, hypertrichosis and pectus deformity. His characteristics were consistent with a diagnosis of WSS, which is a disorder of phenotype heterogeneity. *KMT2A* is widely expressed in the central nervous system (CNS) including the retina and plays crucial role in CNS development (Brightman et al., 2018). The variant p.N1172K in the cysteine-rich CXXC domain (amino acids 1147-1204) is functionally conserved and crucial for binding nonmethylated CpG-containing DNA, contributing to severe symptoms of multisystem involvements (Xu et al., 2018; Luo et al., 2021). The mechanisms of histone modification for growth failure remains to be determined.

The patient with short stature, cataract, and muscle weakness had a *de novo* heterozygous *POLG* pathogenic variant p.Y955H. A previous study had identified another patient with the same variant had a severe early-onset multisystemic involvement with bilateral sensorineural hearing loss, cataract, myopathy, and liver failure (Siibak et al., 2017). This study has demonstrated that the variant p.Y955H in the critical polymerase catalytic domain caused severe mitochondrial DNA (mtDNA) polymerase dysfunction, affecting mtDNA replication with dominant negative effect on DNA synthesis (Bekheirnia et al., 2012; Siibak et al., 2017). Both the patients had similar clinical phenotypes, especially cataracts, suggesting an important role for *POLG* during lens development. The *POLG*-related disorders comprise a continuum of overlapping phenotypes based on biallelic *POLG* pathogenic variants except autosomal dominant progressive external ophthalmoplegia (adPEO,MIM #157640) (Cohen et al., 1993). The disorder is characterized by generalized myopathy, variable degrees of sensorineural hearing loss, axonal neuropathy, ataxia, depression, parkinsonism, hypogonadism and cataracts (Rahman and Copeland, 2019). The patient in our study had some, but not all, of the features of adPEO. Given his young age, his clinical phenotype requires long-term follow-up. Our findings expand the clinical spectrum associated with *POLG*-associated disorders (Bekheirnia et al., 2012). Because of milder phenotypic presentation in early childhood, it’s difficult to recognize and diagnose *POLG*-related disorders in some patients with short stature.

X-linked hypophosphatemic rickets (XLH, MIM #307800) is due to abnormalities of a phosphate-regulating gene homologous to endopeptidase on the X chromosome (PHEX) (Bitzan and Goodyer, 2019). *PHEX*, highly expressed in osteoblasts, osteocytes, and odontoblasts, is a local negative regulator of fibroblast growth factor 23 (*FGF23*). The *PHEX* variant p.W368X might cause PHEX inactivation leading to upregulation of FGF23. FGF23 excess increases inorganic phosphate (Pi) excretion and lowers serum Pi levels by downregulating the renal expression of type IIa and IIc sodium/Pi (Na^+^/Pi) cotransporters (NaPi-IIa and NaPi-IIc) (Yamazaki and Michigami, 2022). In addition, FGF23 reduces the production of 1,25(OH)_2_D, which can increase intestinal Pi absorption by upregulating the expression of the type IIb Na^+^/Pi cotransporter NaPi-IIb, leading to hypophosphatemia (Yamazaki and Michigami, 2022). Hypophosphatemia impairs skeletal mineralization and results in skeletal manifestations of rickets. The proband and his brother were characterized by disproportionate short stature with leg length significantly lower than the sitting height, osteoporosis, severe bone malformations and genu valgus. They had typical chemical findings of XLH: low serum phosphate concentration and elevated alkaline phosphatase. Based on the pathogenesis, XLH is a disorder of FGF23-related hypophosphatemic rickets.

WES has been routinely used for identifying pathogenic variants in genetic disorders and presents a higher diagnostic yield than short stature panels (Li et al., 2022). Recent studies have demonstrated that Trio-WES was an important approach to investigate UPD through analysis of SNP origin from trio sequencing data (Del Gaudio et al., 2020; Chen et al., 2021). Silver-Russell syndrome (SRS, MIM #180860) is a genetically heterogeneous disorder characterized by intrauterine growth retardation, postnatal growth failure, relative macrocephaly at birth, prominent forehead usually with frontal bossing, micrognathia with narrow chin, body asymmetry, clinodactyly, and feeding difficulties (Saal et al., 1993). The proband with SGA (P19) had pre and postnatal growth retardation, wide forehead, triangular face, micrognathia, clinodactyly and low BMI, however, characteristic body asymmetry was obscure. According to the Netchine-Harbison Clinical Scoring System (NH-CSS) clinical criteria, his score was four (Wakeling et al., 2017). Clinical diagnosis of SRS was confirmed by trio-WES, in which mUPD7 was identified as the pathogenic cause. Growth anomalies in mUPD7 were caused by two maternally overexpression of growth suppressor genes and lack of paternally expressed growth promoter genes (Butler, 2020; Zhang et al., 2020). Diagnosis of SRS was challenging as patients with mUPD7 are more likely to have less typical phenotypes than those with loss of methylation on chromosome 11p15 (Bernard et al., 1999; Wakeling, 2011; Wakeling, 2021). UPD evaluation was recommended in Trio-WES analysis for patients who manifested with short stature, especially SGA.

This study suggests that it is important to separate and differentiate between syndromic and isolated short stature patients. Genetic testing has high yield for syndromic patients but low for isolated patients. In this cohort, 70% (14/20) of these patients had negative Trio-WES results. The main reason was that 65% (13/20) patients had isolated short stature, which might be normal variants of growth caused by combination of hundreds of common variants associated with linear growth and environment effects (Wit et al., 2016; Collett-Solberg et al., 2019a; Homma et al., 2019). Comprehensive genetic analysis, such as whole-genome sequencing (WGS) and genome-wide methylation assays, is recommended to explore potential causal variants in deep introns and genetic methylation defects in patients with negative Trio-WES findings (Collett-Solberg et al., 2019a).

Our study highlights the importance of Trio-WES to identify genetic variants and UPD in the molecular diagnosis of syndromic short stature. Except for monogenic causes of short stature, our study identified dual genetic variants in a patient with short stature expanding our knowledge about complex aetiology of growth failure. These molecular defects cause abnormal functions of the cartilage extracellular matrix (*COMP*), paracrine signalling factors (*PHEX*, *FGFR3*-related rickets), transcriptional regulation (*LZTR1*), histone methylation (*KMT2A*), posttranslational modification (*NAA15*), and mtDNA replication and repair (*POLG*) (Andrade et al., 2017). The precise aetiology and signalling pathways of mUPD7 need to be elucidated in further studies. Molecular diagnosis of short stature provides new insights into personalized treatment of growth disorders beyond growth hormone replacement in these individuals.

## Data Availability

The original contributions presented in the study are included in the article, further inquiries can be directed to the corresponding author. The variants *COMP* c.1359delC (p.N453fs*62), *LZTR1* c.509G>A (p.R170Q), *NAA15* c.63T>G (p.Y21X), *KMT2A* c.3516T>A (p.N1172K), *POLG* c.2863T>C (p.Y955H), and *PHEX* c.1104G>A (p.W368X) were submitted to the ClinVar database (http://ncbi.nlm.nlh.nih.gov/clinvar) with the ClinVar variant ID SCV004805897, SCV004805898, SCV004808384, SCV004809195, SCV004809197, SCV004809198.
