# Supplementary material for: Molecular diagnosis of patients with syndromic short stature identified by trio whole-exome sequencing
Source: Front Genet. 2024 Oct 2;15:1399186. doi: 10.3389/fgene.2024.1399186 (PMC11479978; doi:10.3389/fgene.2024.1399186)
Supplement: Supplementary file 1 [file DataSheet1.PDF]

The supplementary table. The primers of Sanger sequencing to confirm the variants.

The variant	Primer (Forward, 5'-3)	Primer (Reverse, 5'-3)	Size(bp)
COMP, c.1359delC p.N453fs*62	CCTCGGTGGGCTAAAGTCAGG	TGTGACAGCGATCAAGACCAGTAA	390
LZTR1, c.509G>A p.R170Q	ATTCTGCTCCACCTTCCAGGGTTT	TGCCCTCAGGGTCCTCATCTG	288
NAA15, c.63T>G, p.Y21X	TTCTGCATCACTCAGTTGTTGGAC	GAGAGGGTCTGTTCTCTCTGGA	417
KMT2A, c.3516T>A, p.N1172K	CCCACACCACAAAATCTAACTGTA	TGAATTCAGTACTCCCTTGGAACT	428
POLG, c.2863T>C, p.Y955H	GGTGTTAAAGTGGATGGGAGAGG	CAGAATGTTCCTGAGCTGCTTTTC	324
PHEX, c.1104G>A, p.W368X	TTGCTGTTAGTTTCCAAAGGCAAAG	TTGGGCTACAAACTCCCCCTG	1603
